# Sleep-related behavior as a factor of anxiety and depression: Mediating role of sleep quality

**DOI:** 10.1192/j.eurpsy.2021.1485

**Published:** 2021-08-13

**Authors:** E. Rasskazova, A. Yavorovskaya

**Affiliations:** 1 Clinical Psychology, Moscow State University, Moscow, Russian Federation; 2 Faculty Of Psychology, Lomonosov Moscow State University, Moscow, Russian Federation

**Keywords:** Anxiety, Depression, sleep-related behavior, sleep quality

## Abstract

**Introduction:**

Although sleep hygiene is a well-studied factor of good sleep (Irish et al., 2015, McNail et al., 2016), less is known about its role in the complaints on anxiety and depression (wither direct or through sleep quality).

**Objectives:**

The aim was to reveal direct and indirect effects of sleep behavior on subjective sleep quality, anxiety and depression.

**Methods:**

174 people aged 17-57 without diagnosed sleep disorders filled the Scale of Behavioral Factors of Sleep Disturbances (Rasskazova, Leonov, 2020), Insomnia Severity Index (Morin, 1993), Hospital Scale of Anxiety and Depression (Zingmond, Snaith, 1983), Beck’s Anxiety and Depression Inventories (Beck, Steer, 1993, Beck et al., 1996).

**Results:**

Taking medications and non-medications before sleep, alcohol, tonic drinks and using gadgets in the evening, delaying bedtime, self-limitations after poor nights, poor adherence to the regimen and postponement of the morning rise were characterized by an indirect effect on anxiety and depressiveness through poor sleep (|β|=0,03-0,24). Self-limiting behavior and delaying the morning rise are associated with higher levels of anxiety and depression, even in the absence of sleep-related complaints (β=0,23-0,34, p<0,01).

**Conclusions:**

Based on the data we suggest that the dysfunctional role of behavior on anxiety and depression is predominantly indirect (through the perpetuation of complaints), but it can also be direct (regardless of complaints of sleep disorders). Research is supported by the Russian Foundation for Basic Research, project No. 20-013-00740.

**Conflict of interest:**

Research is supported by the Russian Foundation for Basic Research, project No. 20-013-00740.

